# Concise Total Synthesis and Antifungal Activities of Fusaric Acid, a Natural Product

**DOI:** 10.3390/molecules25173859

**Published:** 2020-08-25

**Authors:** Bin Bin Huang, Ya Yi Liu, Peng Fei Zhu, Yi Cheng Jiang, Ming-An Ouyang

**Affiliations:** Key Laboratory of Biopesticide and Chemical Biology (Ministry of Education), Fujian Agriculture and Forestry University, Fuzhou 350002, China; bbhuang@fafu.edu.cn (B.B.H.); yayiliu7180@163.com (Y.Y.L.); z15960505490@163.com (P.F.Z.); jiang1658517698@163.com (Y.C.J.)

**Keywords:** total synthesis, 5-butylpicolinic acid, natural product, alkaloid, antifungal activity

## Abstract

The total synthesis of a natural product alkaloid fusaric acid (FA), which exhibits herbicide, fungicide, insecticide and even diverse notable pharmacological activities, was accomplished in four steps using commercially available materials. The synthesis, based on a unified and flexible strategy using 6-bromonicotinaldehyde as a common intermediate, is concise, convergent, practical and can be carried out on a two-gram scale. This approach could be readily applicable to the synthesis of its analogues. In addition, FA had a wide range of inhibitory activities against 14 plant pathogenic fungi in this study, which demonstrated that as a leading compound, and it has great potential to be further developed as an agricultural fungicide.

## 1. Introduction

Plant diseases are the formidable enemy of agricultural production. According to the statistics of the Food and Agriculture Organization of the United Nations (FAO), the crop yield loss caused by plant pathogens is about 10% to 15% of the total output on average annually [1]. The use of fungicides against plant pathogens is a major means of ensuring crop production, so global agriculture has always maintained a strong demand for fungicides on the market. In the early 1970s, with the registration and widespread use of the systemic fungicide benomyl (Benlate), the problem of fungicide resistance became apparent. Now, significant fungicide resistance is a widespread problem in global agriculture. In this silent battlefield of fighting against the development of fungicide resistance, the most important and fundamental weapon that mankind can grasp is to accelerate the research of new structural compounds that can be used for the agricultural field, and further find new target sites and modes of action [2].

Fusaric acid (FA), chemically known as 5-butylpicolinic acid, is a natural product alkaloid, with low to moderate toxicity [3], isolated by Yabuta T. in 1934 from the secondary metabolites of *Fusarium heterosporium* that parasitizes rice [4]. A large number of research data showed that FA has diverse notable pharmacological activities, ranging from anti-hypertensive [5,6] and anti-viral [7,8] activities to anti-cancer effects [9,10] and the treatment of Parkinson’s disease [11,12]. As a kind of phytotoxin, FA and its derivatives also exhibited marked herbicidal activity [13,14]. It also has certain insecticidal activity as an important insect phenoloxidase inhibitor [15,16]. In addition, FA showed strong antifungal activity in both medical and agricultural fields [17,18].

**Scheme 1:**
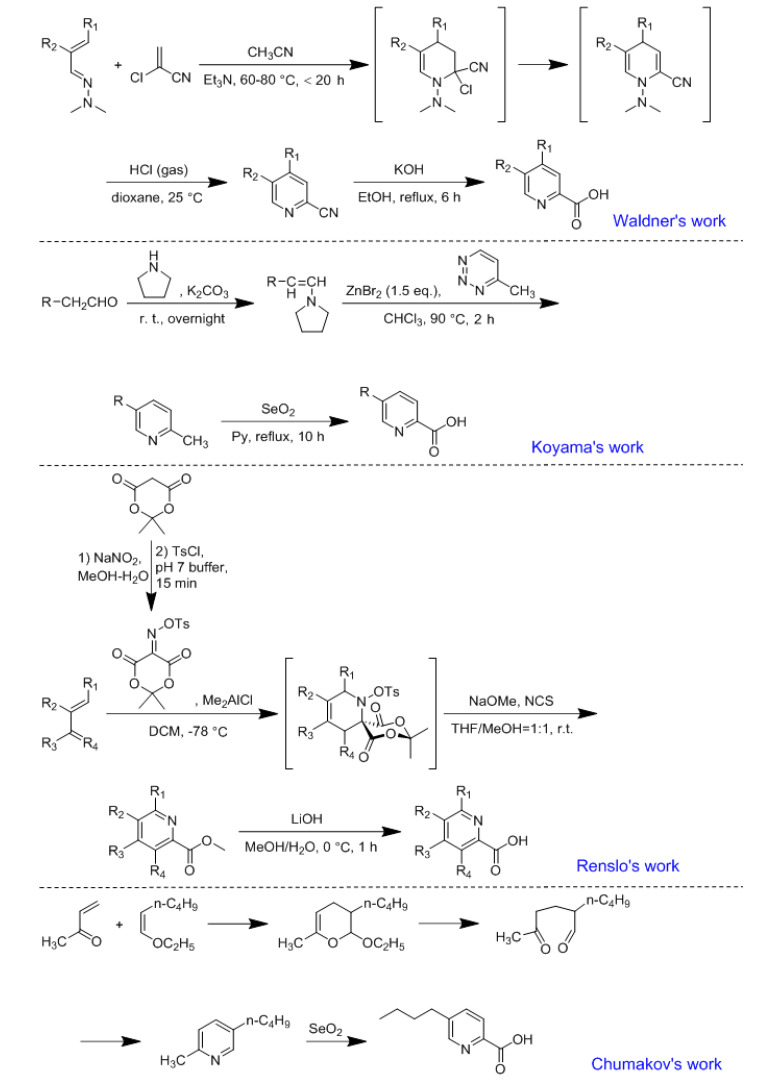
Previous work for total synthesis of fusaric acid (FA) by constructing pyridine ring via Diels-Alder type reaction.

**Scheme 2:**
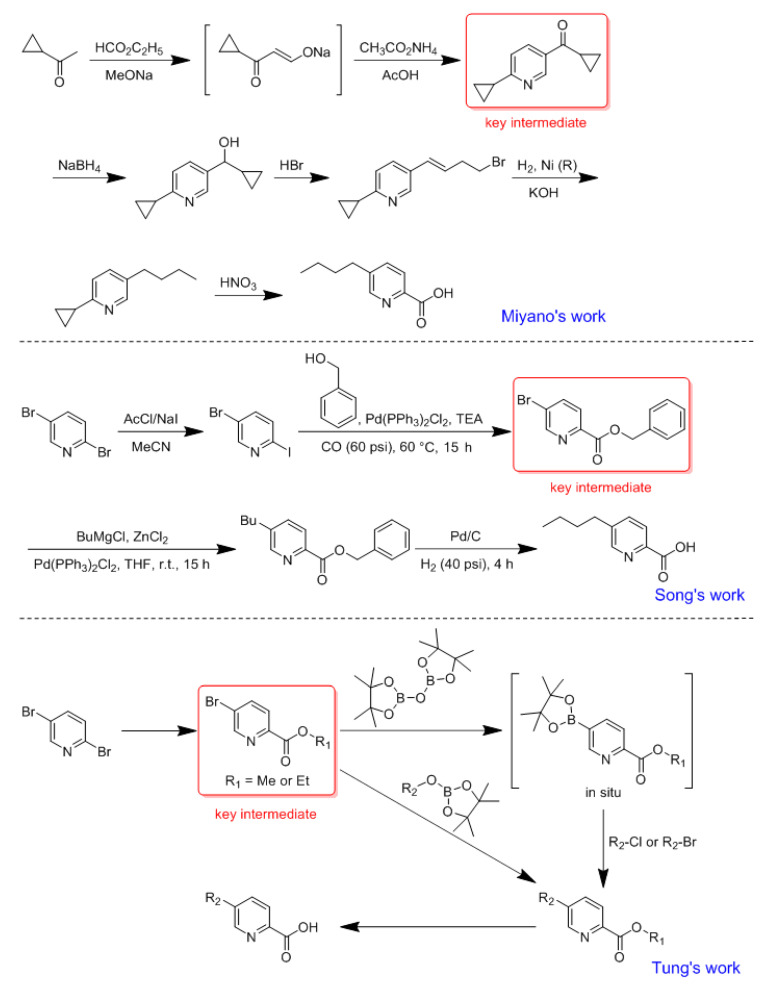
Previous work for total synthesis of FA by elaborating substituted pyridine intermediate.

FA has been discovered for nearly 90 years, during which many synthetic approaches have been developed. Generally, according to the methods of establishing the target structures, these synthetic approaches can be classified into two categories: by constructing a pyridine ring via Diels-Alder type reactions (Scheme 1) [19,20,21,22] and by elaborating substituted pyridine intermediates (Scheme 2) [23,24,25,26,27,28]. The reported syntheses using Diels–Alder reactions either must be carried out under harsh conditions, or substances such as selenium dioxide (SeO_2_) [21,23,29], gaseous hydrogen chloride (HCl) [19], potassium permanganate (KMnO_4_) [24] and magnesium amalgam [24], which are both toxic to human body and harmful to the environment, were used, and these methods are not suitable for large-scale synthesis. Substituted pyridine intermediates designed and elaborated can readily facilitate the synthesis of FA and its analogs. Song et al. [27] prepared the key intermediate benzyl 5-bromopicolinate from raw material 2,5-dibromopyridine, and then synthesized FA through Negishi coupling, catalytic hydrogenation reaction and recrystallization purification. Tung et al. [28] prepared the key intermediates methyl (or ethyl) 5-bromopicolinate, then synthesized FA and its analogies via two-step reaction of Suzuki coupling and hydrolysis. We have practiced these two routes and found that the yield of Negishi and Suzuki coupling reactions was not satisfactory (<15%) due to the ester moieties. Hence, a more practical and scalable approach with an easily accessible reagent is still highly desirable to synthesize FA and its analogues to facilitate further antifungal activity evaluations.

**Scheme 3:**
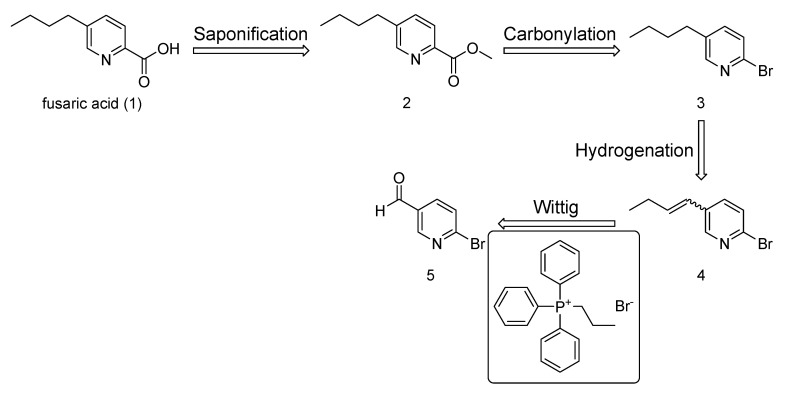
Synthetic plan for FA.

We envisioned that the sequential reactions, such as Wittig and carbonylation reactions, at C(2) and C(5) positions of the pyridine ring would enable us to establish a concise and flexible route to a range of FA and its derivatives from a template 6-bromonicotinaldehyde, the commercially available compound (Scheme 3).

The objective of this study was to develop a concise, practical and scalable synthesis of FA and to facilitate further determinations of the FA inhibitory activities against several plant pathogenic fungi. In the meantime, we wish to reveal the great potential which FA has for further developing as an agricultural fungicide.

## 2. Results and Discussion

### 2.1. Total Synthesis of FA

**Scheme 4:**
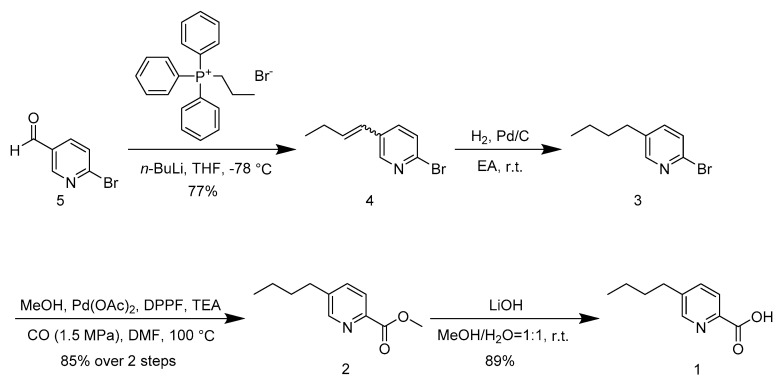
Total synthesis of FA.

Our synthesis (Scheme 4) began with the preparation of 2-bromo-5-(but-1-en-1-yl)pyridine (**4**) from commercially available 6-bromonicotinaldehyde (**5**) and *n*-propyltriphenyl-phosphonium bromide by using the Wittig reaction [30], followed by a hydrogenation reaction [31] to afford 2-bromo-5-butylpyridine (**3**). Once 2-bromo-5-butylpyridine (**3**) was in hand, a carbonylation reaction [32] was carried out immediately and methyl 5-butylpicolinate (**2**) was obtained. To our delight, all the reactions had run very well and afforded the expected end-product FA (**1**) in high yield after a saponification reaction [33]. It is noteworthy that the synthetic route is reliable, practical and gram scale for the desired FA (**1**), of which the total yield was 58% over only four steps and all spectroscopic data for our synthetic samples were in good agreement with those reported for the corresponding natural products.

Another simple method of preparing carboxylic acid by halogenated hydrocarbon is to introduce the cyano group through the reaction of halogenated hydrocarbon [34] or diazonium salt [35] with inorganic cyanide before hydrolysis [36]. However, the use of hazardous chemicals such as diazonium salts or inorganic cyanide is unavoidable. In this study, the methyl ester of FA was prepared by carbonylation reaction of halogenated hydrocarbons. Although the reaction requires palladium catalyst and pressure, it is a feasible process route for practical and scalable preparation at one time, especially if industrial production was required.

Additionally, since *n*-butyl lithium was used in the Wittig reaction, the reaction should be carried out at −78 °C, which may have adverse effects on the scale-up production. This step of the reaction was also explored preliminarily. When anhydrous dimethyl formamide (DMF) was used as solvent and *n*-butyl lithium was replaced by sodium hydride (NaH), the dehydrogenation reaction could be carried out at −10 °C, and the yield of 2-bromo-5-(but-1-en-1-yl)pyridine (**4**) was about 65%.

### 2.2. In Vitro Inhibitory Activities of FA against 14 Plant Pathogenic Fungi

The results in Table 1 show that FA had a wide range of inhibitory activities against 14 plant pathogenic fungi, although the inhibition rates of FA against *Valsa mal* and *Alternaria longipes* at 200 μg/mL were only 12.93 ± 1.18% and 28.57 ± 2.04%, respectively. Moreover, FA had the best inhibitory activities against *Thanatephorus cucumeris*, *Colletotrichum higginsianum* and *Colletotrichum gloeosporioides*, whose 50% effective concentration (EC_50_) were 22.42, 31.67 and 90.80 μg/mL, respectively.

## 3. Materials and Methods

### 3.1. General

Reactions were carried out in flame- or oven-dried glassware under an argon atmosphere, unless otherwise noted. Tetrahydrofuran (THF) was freshly distilled before use from sodium using benzophenone as an indicator. All other anhydrous solvents were dried over 3 or 4 Å molecular sieves. The solvents used in workup, extraction, and column chromatography were used as received from commercial suppliers without prior purification. Reactions were magnetically stirred and monitored by thin layer chromatography (TLC, 0.25 mm) on pre-coated silica gel plates produced by Yantai Jiangyou silica gel Development Co., Ltd. (Yantai, Shandong Province, China). Flash chromatography was performed with silica gel 60 (particle size 0.040–0.062 mm) supplied by Qingdao Haiyang Chemical Co., Ltd. (Qingdao, Shandong Province, China). High resolution mass spectra were measured at Keecloud Mass Spectrometry Service Company, (Shanghai, China), on an Thermo Scientific LTQ Orbitrap XL system, Waltham, MA, USA. ^1^H and ^13^C-NMR spectra were recorded on a Bruker AVIII-400 spectrometer (400 MHz for ^1^H, 100 MHz for ^13^C), (Rheinstetten, German). Chemical shifts were reported in parts per million (ppm) as values relative to the internal chloroform (7.26 ppm for ^1^H and 77.16 ppm for ^13^C). Abbreviations for signal coupling are as follows: s, singlet; d, doublet; t, triplet; q, quartet; m, multiplet. The visualization of NMR spectra could be found in the Supplementary Materials.

### 3.2. Total Synthesis of FA

#### 3.2.1. Synthesis of 2-Bromo-5-(but-1-en-1-yl)pyridine (**4**)

To a solution of *n*-propyltriphenyl-phosphonium bromide (9.25 g, 24.0 mmol) in anhydrous THF (25 mL) was added *n*-butyllithium (13.75 mL, 22.0 mmol, 1.6 M in hexane) dropwise at −78 °C and under an argon atmosphere. The reaction mixture was stirred for 15 min and then heated to 0 °C for at least 1 h. After the reaction mixture was cooled to −78 °C, the solution dissolved 6-bromonicotinaldehyde (**5**) (3.72 g, 20.0 mmol) in anhydrous THF (25 mL) was added. It was stirred for 15 min and then heated to room temperature (RT); the reaction mixture was stirred at RT until the complete consumption of starting material was observed by TLC (3 h). Aqueous saturated ammonium chloride (NH_4_Cl) solution (10 mL) was added under vigorous stirring to quench the reaction. The volatiles (mainly THF) were removed under reduced pressure and the aqueous phase was extracted with dichloromethane (3 × 20 mL). The combined organic fractions were washed with water and brine, dried over anhydrous sodium sulfate (Na_2_SO_4_) and evaporated under reduced pressure. The residue was purified by flash column chromatography on silica gel using eluents (petroleum ether/ethyl acetate = 20/1) to afford 2-bromo-5-(but-1-en-1-yl)pyridine (**4**) as a colorless oil (3.28 g, 77% yield). Major diastereomer. ^1^H-NMR (400 MHz, CDCl_3_) δ 8.26 (s, 1H), 7.45–7.43 (m, 2H), 6.26 (dt, *J* = 11.6, 1.5 Hz, 1H), 5.83 (dt, *J* = 11.6, 7.4 Hz, 1H), 2.31–2.25 (m, 2H), 1.06 (t, *J* = 7.5 Hz, 3H). ^13^C-NMR (100 MHz, CDCl_3_) δ 150.1, 139.7, 138.4, 138.1, 132.7, 127.6, 123.5, 22.1, 14.3. Minor diastereomer. ^1^H-NMR (400 MHz, CDCl_3_) δ 8.29 (d, *J* = 2.5 Hz, 1H), 7.53 (dd, *J* = 8.1, 2.5 Hz, 1H), 7.40 (d, *J* = 8.1, 1H), 6.39–6.29 (m, 2H), 2.25–2.21 (m, 2H), 1.11–1.10 (m, 3H). ^13^C-NMR (100 MHz, CDCl_3_) δ 148.1, 139.7, 136.3, 135.1, 133.0, 127.9, 124.1, 26.3, 13.4.

#### 3.2.2. Synthesis of 2-Bromo-5-butylpyridine (**3**)

A round bottom flask was charged with 2-bromo-5-(but-1-en-1-yl)pyridine (**4**) (3.28 g, 15.5 mmol), then evacuated and backfilled with argon. After ethyl acetate (30 mL) and 10% Pd/C (1.65 g, 1.55 mmol) were added under argon, the argon in the round bottom flask was replaced by hydrogen for at least 10 min. It was stirred at RT under a hydrogen atmosphere until the complete consumption of starting material was observed by TLC (15 min). The resulting suspension was filtered through a compacted diatomaceous earth layer eluting with dichloromethane (3 × 20 mL). The combined filtrate was collected and evaporated under reduced pressure to afford a colorless oil. It was used directly as crude (**3**) in the next step immediately.

#### 3.2.3. Synthesis of Methyl 5-butylpicolinate (**2**)

The crude (**3**), palladium (II) acetate (0.067 g, 0.30 mmol), 1,1′-Bis(diphenylphosphino)ferrocene (DPPF; 0.333 g, 0.60 mmol), trimethylamine (TEA; 2.98 g, 29.5 mmol) and methanol (12 mL, 294.8 mmol) were dissolved in DMF (30 mL) and the solution was stirred under carbon monoxide (CO; 1.5 MPa) at 100 °C overnight. The reaction mixture was cooled to RT before most of the volatile components were removed under reduced pressure. Saturated sodium chloride (NaCl) solution (100 mL) was added and then the mixture was shaken vigorously. The solution was extracted with ethyl acetate (3 × 30 mL), and the combined organic layers were washed with water and brine, dried (Na_2_SO_4_), filtered and concentrated by evaporating under reduced pressure. The resulting residue was purified by flash chromatography on silica gel using eluents (petroleum ether/ethyl acetate = 10/1) to afford the desired methyl 5-butylpicolinate (**2**) (2.56 g, 85% yield over 2 steps) as a colorless oil. ^1^H-NMR (400 MHz, CDCl_3_) δ 8.55 (d, *J* = 1.8 Hz, 1H), 8.05 (d, *J* = 8.0 Hz, 1H), 7.64 (dd, *J* = 8.0, 1.8 Hz, 1H), 3.99 (s, 3H), 2.68 (t, *J* = 7.6 Hz, 2H), 1.67–1.56 (m, 2H), 1.42–1.30 (m, 2H), 0.93 (t, *J* = 7.3 Hz, 3H). ^13^C-NMR (100 MHz, CDCl_3_) δ 165.9, 150.1, 145.6, 142.4, 136.8, 125.1, 52.9, 33.1, 32.9, 22.3, 13.9.

#### 3.2.4. Synthesis of FA (**1**)

Methyl 5-butylpicolinate (**2**) (2.56 g, 13.2 mmol) was mixed with lithium hydrate (LiOH; 1.58 g, 66.0 mmol) in a 30 mL methanol/water (*v*/*v* = 1:1) mixed solvent, and then it was stirred at RT. After TLC analysis indicated complete consumption of the starting material (1 h), the volatiles (mainly methanol) were removed under reduced pressure. The pH of the resulting suspension was adjusted to about 2 with 1.0 M HCl. The aqueous phase was extracted with dichloromethane until no product was observed in the aqueous phase by TLC. The combined organic layers were dried over anhydrous Na_2_SO_4_, filtered and evaporated under reduced pressure. The desired FA (*1*) was obtained as a white solid (2.12 g, 89% yield). Mp = 99–101 °C. (lit. 99–100.5 °C) [22] ^1^H-NMR and ^13^C-NMR of the synthetic material were identical to those reported for the natural product [22]: ^1^H-NMR (400 MHz, CDCl_3_) δ 10.79 (s, 1H), 8.59 (s, 1H), 8.15 (d, *J* = 7.8 Hz, 1H), 7.73 (d, *J* = 7.5 Hz, 1H), 2.70 (t, *J* = 7.5 Hz, 2H), 1.73–1.51 (m, 2H), 1.45–1.25 (m, 2H), 0.92 (t, *J* = 7.3 Hz, 3H). ^13^C-NMR (100 MHz, CDCl_3_) δ 165.8, 148.1, 145.2, 143.1, 138.4, 124.4, 33.1, 32.9, 22.4, 13.9. HRMS (ESI) *m*/*z* calculated for C_10_H_13_NO_2_ [M + H]^+^ 180.10191, found 180.10147.

### 3.3. In Vitro Inhibitory Activities of FA against 14 Plant Pathogenic Fungi

The antifungal activities were evaluated against 14 pathogenic fungi, namely, *C. higginsianum*, *H. maydis*, *P. grisea*, *T. cucumeris*, *B. cinerea*, *G. catenulatum*, *C. gloeosporioides*, *P. aphanidermatum*, *V. mal*, *M. arachidis*, *S. sclerotiorum*, *P. capsici*, *R. solani*, and *A. longipes*, by the poison plate technique [37,38,39]. FA was dissolved in chromatographic grade methanol (2 mL) to prepare a 10 mg/mL stock solution. A proper amount of stock solution was precisely transferred with a pipette, and mixed with sterilized and melted potato dextrose agar (PDA; 49 mL). The corresponding amount of chromatographic grade methanol and sterile distilled water were added to make the total volume of 50 mL with 2% methanol content. FA was tested at five double-declining concentrations (e.g., 200, 100, 50, 25, and 12.5 μg/mL). Meantime, all fungi were cultivated in PDA at 28.0 ± 0.5 °C for 5 days in darkness to make new mycelium for the identification of antifungal activity. Then, mycelia dishes of approximately 5 mm diameter were cut from the culture medium. A mycelium was obtained using a germ-free inoculation needle and inoculated reversely in the center of the PDA plate aseptically. The inoculated plates were incubated at 28.0 ± 0.5 °C for 2–7 days in darkness. Chromatographic-grade methanol in sterile distilled water served as the control. At least three biological replicates and controls were used for each concentration. Radial growth of the fungal colonies was measured, and the data were statistically analyzed. Inhibitory activities of FA in vitro on these fungi were calculated by the formula I (%) = [(C − T)/(C − 5.00)] × 100, where C represents the diameter of fungal growth on untreated PDA (blank PDA with 2% methanol), T represents the diameter of fungal growth on treated PDA, and I represents the inhibition rate. Finally, the corresponding toxic regression equation, EC_50_, 95% confidence interval and determination coefficient were obtained by using the software package SPSS 22.0.

## 4. Conclusions

In summary, we have developed a total synthesis of naturally occurring alkaloid FA in four steps with a 58% yield from 6-bromonicotinaldehyde (**5**), by taking advantage of Wittig reaction, hydrogenation, carbonylation and saponification. The synthesis is concise, convergent, practical and can be carried out on a two-gram scale. Since this synthesis is based on a unified and flexible strategy using 6-bromonicotinaldehyde (**5**) as a common intermediate, it is readily applicable to analogue synthesis. Furthermore, the results from in vitro antifungal activities of FA reveal that FA, as a leading compound, has great potential to be further researched and developed as an agricultural fungicide. Further exploration of the strategy for the generation of novel analogues for biological evaluation is ongoing in our laboratory. As a kind of phytotoxin, FA can cause some plants to wilt or inhibit root elongation, such as bananas [40,41], tomatoes [13], tobacco [42,43], maize [44], and watermelons [45,46], etc. In order to ensure the plants of agricultural interest being safe, novel FA analogues for further biological evaluation should be evaluated as to whether the analogues might cause phytotoxicity at an effective dose. Relevant study work is also ongoing in our laboratory.

## Figures and Tables

**Table 1:** Toxicity of FA against 14 plant pathogenic fungi.

Plant Pathogenic Fungus	EC_50_ (95% CL) (μg/mL)	Toxic Regression Equation	Determination Coefficient
*Colletotrichum higginsianum*	31.67 (26.72–37.30)	y = 1.954x + 2.068	0.9462
*Helminthosporium maydis*	130.25 (115.68–149.24)	y = 1.666x + 1.476	0.9703
*Pyricularia grisea*	120.39 (107.26–137.04)	y = 1.684x + 1.497	0.9999
*Thanatephorus cucumeris*	22.42 (10.20–34.24)	y = 1.334x + 3.199	0.9417
*Botrytis cinerea*	338.10 (258.80–484.10)	y = 1.166x + 2.051	0.9806
*Gliocladium catenulatum*	241.31 (193.89–367.29)	y = 2.726x − 1.494	0.9990
*Colletotrichum gloeosporioides*	90.80 (60.23–171.70)	y = 0.969x + 3.103	0.9745
*Pythium aphanidermatum*	173.46 (150.11–210.84)	y = 1.616x + 1.382	0.9897
*Valsa mal*	–		
*Mycosphaerella arachidis*	187.60 (174.98–203.80)	y = 3.941x − 3.959	0.9530
*Sclerotinia sclerotiorum*	190.68 (158.70–262.93)	y = 2.703x − 1.163	0.9969
*Phytophthora capsici*	170.89 (147.58–208.44)	y = 1.570x + 1.494	0.9812
*Rhizoctonia solani*	221.17 (174.97–358.79)	y = 2.695x − 1.318	0.9893
*Alternaria longipes*	–		

## Supplementary Materials

The following are available online. Copies of ^1^H and ^13^C of new compounds.
