# Supplementary material for: Concise Total Synthesis and Antifungal Activities of Fusaric Acid, a Natural Product
Source: Molecules. 2020 Aug 25;25(17):3859. doi: 10.3390/molecules25173859 (PMC7503603; doi:10.3390/molecules25173859)

2-bromo-5-(but-1-en-1-yl)pyridine (4)： **Minor diastereomer.** ^{1}H NMR (400 MHz, $CDCl_3$) δ 8.29 (d, *J* = 2.5 Hz, 1H), 7.53 (dd, *J* = 8.1, 2.5 Hz, 1H), 7.40 (d, *J* = 8.1, 1H), 6.39 – 6.29 (m, 2H), 2.25 – 2.21 (m, 2H), 1.11 – 1.10 (m, 3H). **Major diastereomer.** ^{1}H NMR (400 MHz, $CDCl_3$) δ 8.26 (s, 1H), 7.45 – 7.43 (m, 2H), 6.26 (dt, *J* = 11.6, 1.5 Hz, 1H), 5.83 (dt, *J* = 11.6, 7.4 Hz, 1H), 2.31 – 2.25 (m, 2H), 1.06 (t, *J* = 7.5 Hz, 3H).

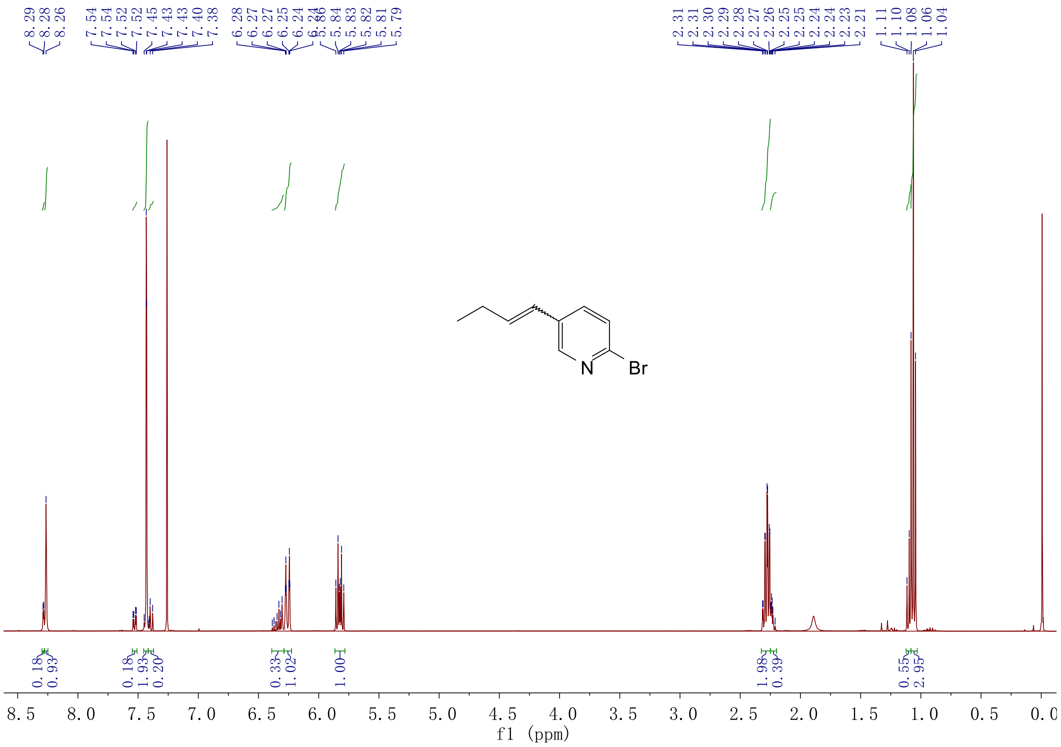

2-bromo-5-(but-1-en-1-yl)pyridine (4)： **Minor diastereomer.** ^{13}C NMR (100 MHz, $CDCl_3$) δ 148.1, 139.7, 136.3, 135.1, 133.0, 127.9, 124.1, 26.3, 13.4. **Major diastereomer.** ^{13}C NMR (100 MHz, $CDCl_3$) δ 150.1, 139.7, 138.4, 138.1, 132.7, 127.6, 123.5, 22.1, 14.3.

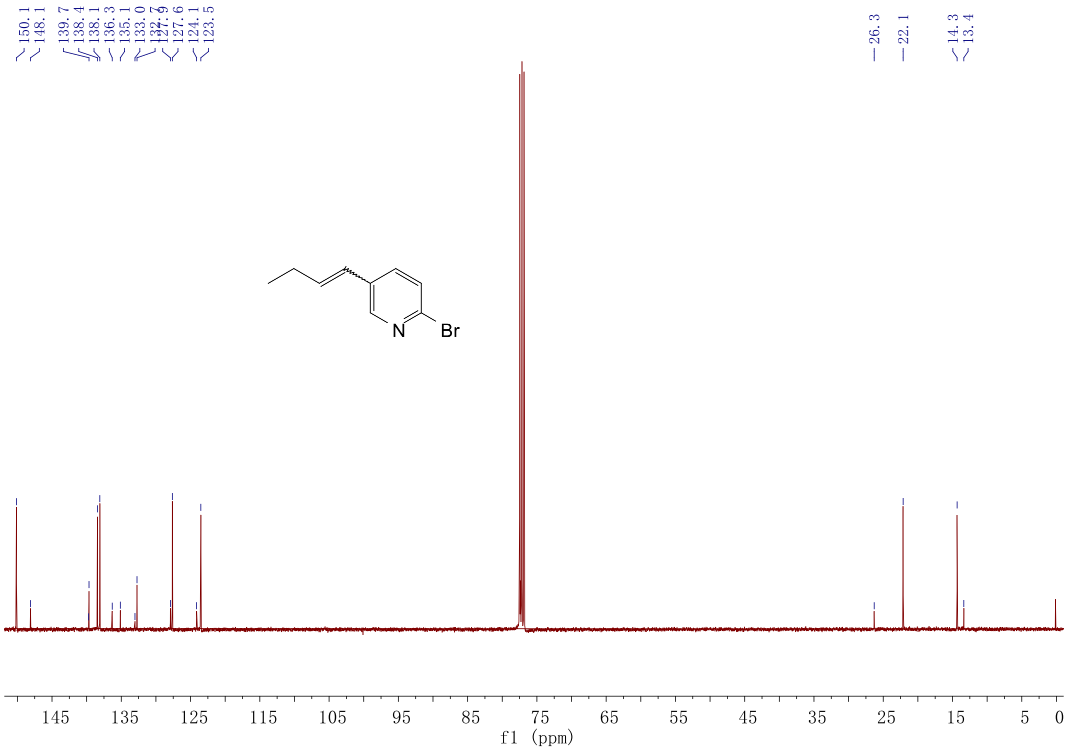

methyl 5-butylpicolinate (2): ^{1}H NMR (400 MHz, $CDCl_3$) δ 8.55 (d, *J* = 1.8 Hz, 1H), 8.05 (d, *J* = 8.0 Hz, 1H), 7.64 (dd, *J* = 8.0, 1.8 Hz, 1H), 3.99 (s, 3H), 2.68 (t, *J* = 7.6 Hz, 2H), 1.67 – 1.56 (m, 2H), 1.42 – 1.30 (m, 2H), 0.93 (t, *J* = 7.3 Hz, 3H).

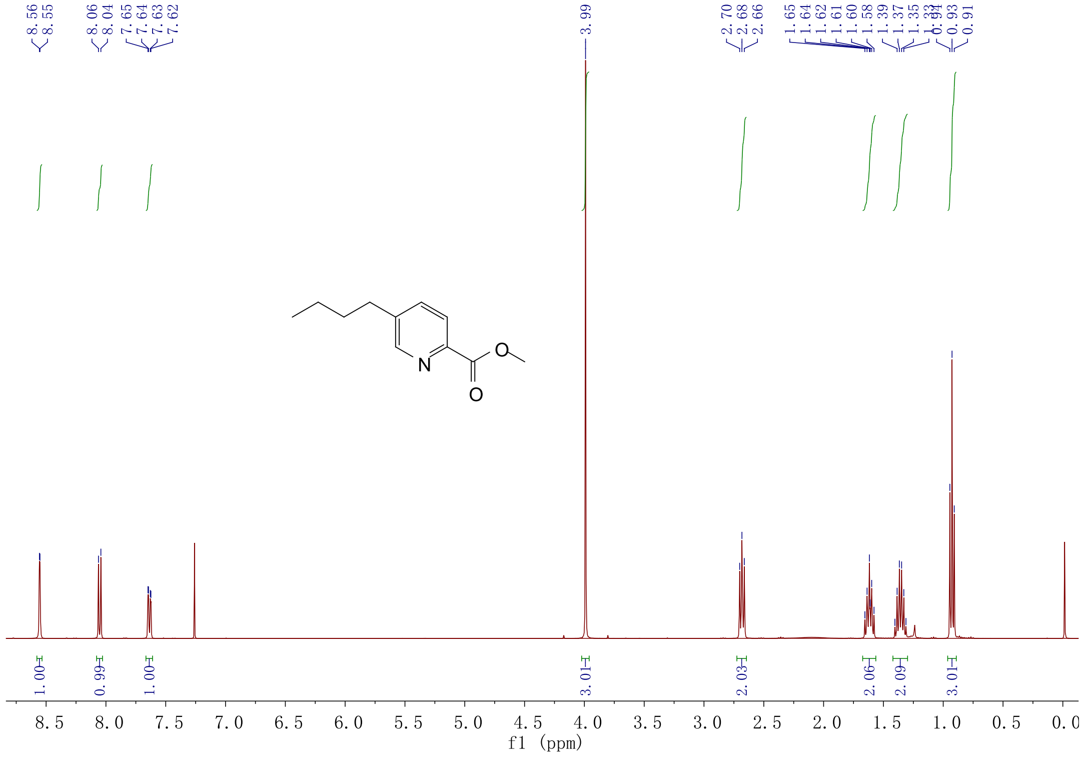

methyl 5-butylpicolinate (2): ^{13}C NMR (100 MHz, $CDCl_3$) δ 165.9, 150.1, 145.6, 142.4, 136.8, 125.1, 52.9, 33.1, 32.9, 22.3, 13.9.

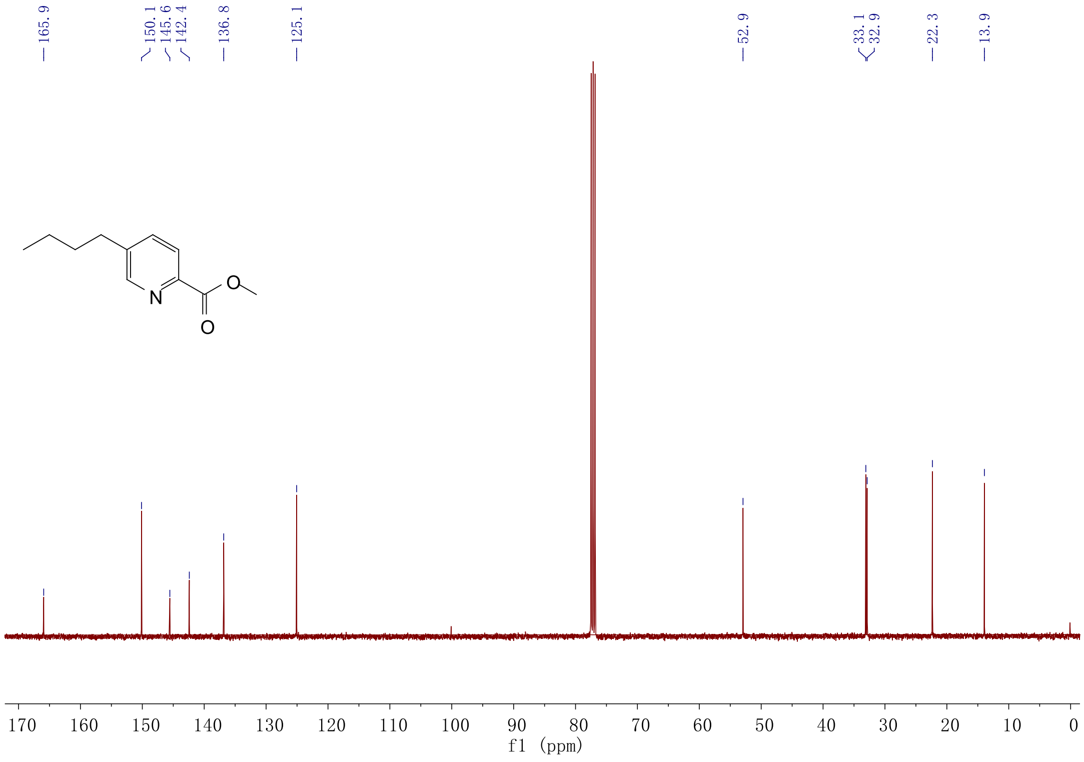

Fusaric Acid： ^{1}H NMR (400 MHz, $CDCl_3$) δ 10.79 (s, 1H), 8.59 (s, 1H), 8.15 (d, J = 7.8 Hz, 1H), 7.73 (d, J = 7.5 Hz, 1H), 2.70 (t, J = 7.5 Hz, 2H), 1.73 – 1.51 (m, 2H), 1.45 – 1.25 (m, 2H), 0.92 (t, J = 7.3 Hz, 3H).

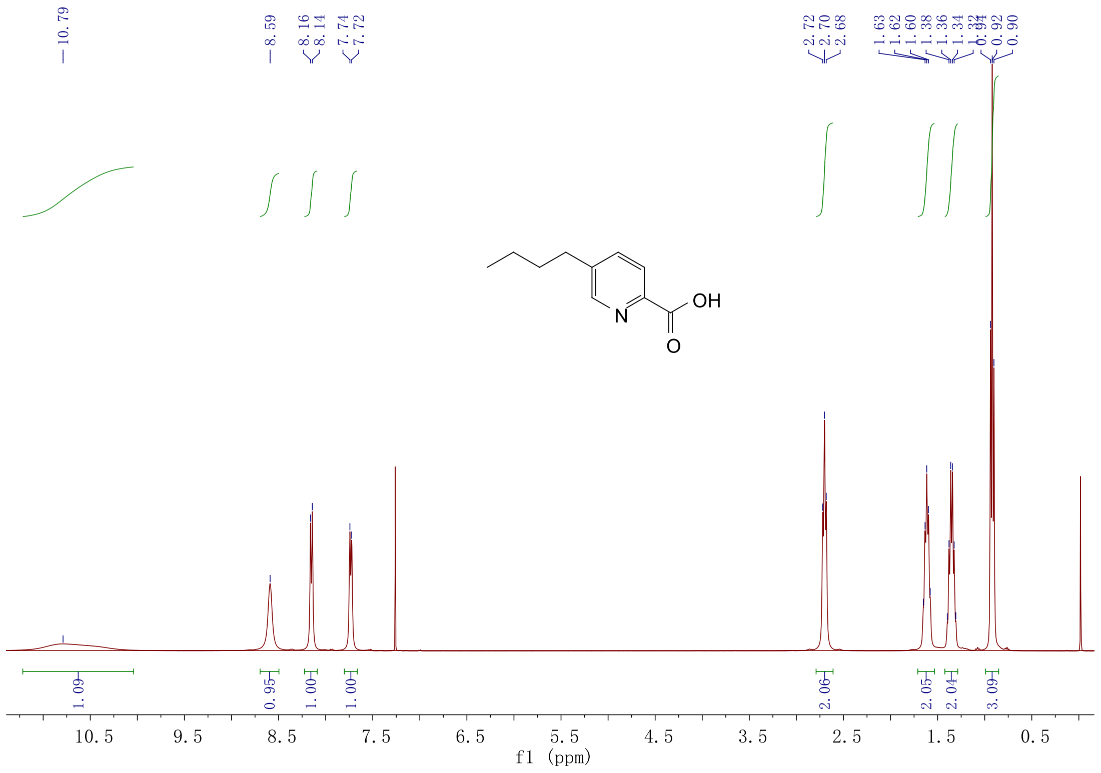

Fusaric Acid： ^{13}C NMR (100 MHz, $CDCl_3$) δ 165.8, 148.1, 145.2, 143.1, 138.4, 124.4, 33.1, 32.9, 22.4, 13.9.

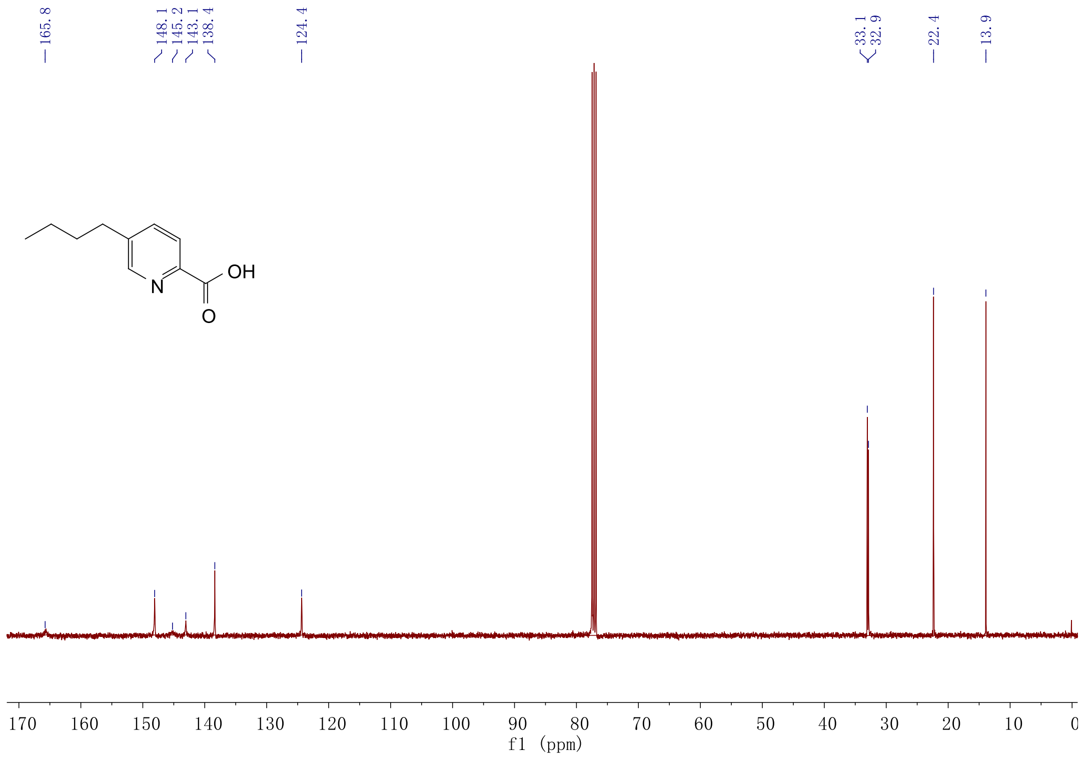

Fusaric Acid: HRMS (ESI) m/z calculated for $C_{10}H_{13}NO_2$ $[M+H]^+$ 180.10191, found 180.10147.

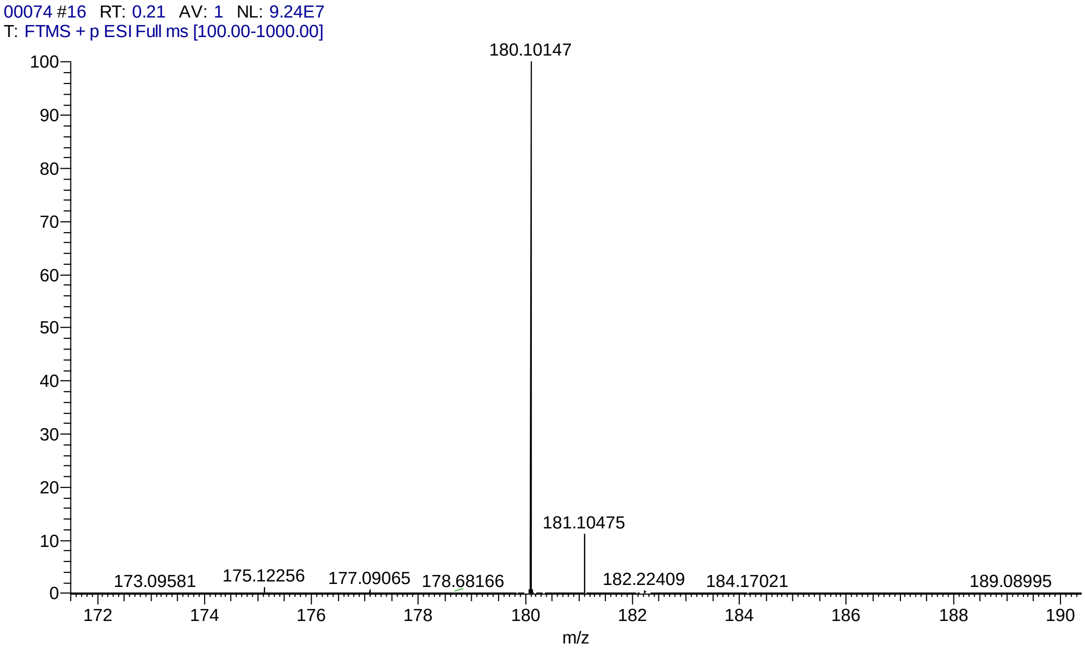

Supplement: Supplementary file 1 [file molecules-25-03859-s001.pdf]
